# Cost effectiveness of pharmacogenetic-guided clozapine administration based on risk of HLA variants in Japan and the UK

**DOI:** 10.1038/s41398-021-01487-4

**Published:** 2021-07-07

**Authors:** Kohei Ninomiya, Takeo Saito, Tomo Okochi, Satoru Taniguchi, Ayu Shimasaki, Rei Aoki, Takeo Hata, Taisei Mushiroda, Tetsufumi Kanazawa, Masashi Ikeda, Nakao Iwata

**Affiliations:** 1 Department of Psychiatry, Fujita Health University School of Medicine, Toyoake, Aichi Japan; 2 Department of Pharmacy, Osaka Medical College Hospital, Takatsuki, Osaka Japan; 3 Laboratory for Pharmacogenomics, RIKEN Center for Integrative Medical Sciences, Yokohama, Japan; 4 Department of Neuropsychiatry, Osaka Medical College, Takatsuki, Osaka Japan

**Keywords:** Schizophrenia, Predictive markers

## Abstract

Pharmacogenetics/pharmacogenomics have enabled the detection of risk of human leukocyte antigen (HLA) variants for clozapine-induced agranulocytosis/granulocytopenia (CIAG). To apply this evidence to the clinical setting, we compared the cost-effectiveness of the proposed “HLA-guided treatment schedule” and the “current schedule” being used in Japan and the United Kingdom (UK) (absolute neutrophil count (ANC) cutoff at 1500/mm^3^); in the “HLA-guided treatment schedules,” we considered a situation wherein the HLA test performed before clozapine initiation could provide “a priori information” by detecting patients harboring risk of HLA variants (HLA-B*59:01 and “HLA-B 158T/HLA-DQB1 126Q” for Japanese and Caucasian populations, respectively), a part of whom can then avoid CIAG onset (assumed 30% “prevention rate”). For the primary analysis, we estimated the incremental cost-effectiveness ratio (ICER) of “HLA-guided treatment schedule” and “current schedule” used in Japan and the UK, using a Markov model to calculate the cost and quality-adjusted life years (QALYs) over a 10-year time period. Furthermore, as an explorative analysis, we simulated several situations with various ANC cutoffs (1000/mm^3^ and 500/mm^3^) and plotted the cost/QALYs for each option to identify the best, or estimate the next best candidate option applicable in actual clinical settings. The primary probabilistic analysis showed that the “HLA-guided treatment schedule” was more cost effective than the “current schedule”; the ICER was £20,995 and £21,373 for the Japanese and the UK populations, respectively. Additional simulation revealed that the treatment option of ANC cutoff at 500/mm^3^ without HLA screening was the most cost-effective option; however, several options may be candidates to break away from the “current schedule” of ANC cutoff at 1500/mm^3^. Owing to its cost-effectiveness, we propose such pharmacogenetic-guided/pharmacogenomic-guided clozapine treatment for use in the real-world setting, which provides key information for optimization of clinical guidelines for high-risk patients for gradual change of clozapine treatment schedule under the safety consideration.

## Introduction

Schizophrenia is a chronic mental disorder with a global prevalence of approximately 1% [1]. The outcomes in schizophrenia vary considerably, ranging from full recovery to severe deficit; 1/3rd of the patients reportedly have treatment-resistant schizophrenia (TRS) [2, 3]. For TRS patients, clozapine (CLZ) is one of the most effective and well-established antipsychotics, however, CLZ induces serious adverse effects such as CLZ-induced agranulocytosis/granulocytopenia (CIA/CIG: collectively known as CIAG). In fact, in the 1970s, the high prevalence of sepsis related to CIAG was a major concern of the CLZ treatment. However, after the introduction of the registry-based scheme for CLZ prescription, the Clozaril Patient Monitoring Service (CPMS), the mortality attributed to sepsis considerably reduced. This is because it is mandatory for psychiatrists to monitor the white blood cell (WBC) count, and absolute neutrophil count (ANC) throughout the CLZ treatment as per the CPMS protocols: patients newly prescribed CLZ must have their WBC count and ANC tested frequently (weekly, every 2 weeks, or monthly). If the WBC count is <3000/mm^3^ or the ANC is 1500/mm^3^, prescription of the drug is no longer permitted in many countries, including Japan and the United Kingdom (UK).

Although CPMS helps to prevent the onset of CIAG, these adverse effects continue to have negative impacts on the prescription attitude of psychiatrists or the perception of patients owing to the fear of potential sepsis (albeit rare), decreasing the quality of life (QOL) via frequent monitoring and other methods. Therefore, detecting the mechanisms of CIAG and extracting high-risk patients a prior is warranted; in line with this, several pharmacogenetic/pharmacogenomic (PGt/PGx) studies have been conducted [4]. Most studies detected the human leukocyte antigen (HLA) as a risk for CIAG in the Japanese [5] and Caucasian subjects [6]. However, HLA screening is insufficient for direct clinical use because of (1) its low sensitivity (HLA-B*59:01: sensitivity = 24% for Japanese population [5]; HLA-B 158T and HLA-DQB1 126Q: sensitivity = 36% for Caucasian population [6]) and (2) limited clinical resources (i.e., lack of prospective clinical studies).

Therefore, for emphasizing the PGt/PGx findings, a more feasible protocol or supporting data applicable in real clinical setting is essential: one potential simulation is the PGx-guided cost-effectiveness analysis (CEA). In a recent paper [7], the authors examined the cost-effectiveness of the PGx-guided (i.e., single nucleotide polymorphisms [SNPs: HLA-B 158T and HLA-DQB1 126Q]) strategies and showed that the current monitoring schedule might not be more cost-effective than a new schedule (if patients harbor HLA risk, they receive ANC monitoring, but if patients do not have, they do not receive the monitoring). However, it is noteworthy that the impact on incremental cost-effectiveness ratio (ICER) due to the genetic tests was not large because the ICER was USD$3,900,000 for a 3-year horizon (base scenario = their “new schedule”). Also, the model in that study assumed that the patients without the risk SNPs were not scheduled even for regular blood monitoring. Therefore, we presumed that most psychiatrists would hesitate to apply this schedule; psychiatrists always prefer to prevent the adverse effects of treatment, even if it requires the use of “non-cost-effective” tools when there is little possibility of establishing the patients’ risk. In that study, they also modeled another schedule, where patients with risk HLAs received substitute drugs without ANC monitoring. However, this revealed less cost-effectiveness due to decreasing quality-adjusted life years (QALYs) and higher cost by substitute treatment, concluding that the “substitution” schedule was not recommended for clinical use. Therefore, although their findings did not show robust evidence whether the PGx-guided schedules must be used in the clinical setting, the following main finding of this paper is substantially important: “genotype-guided blood sampling before CLZ initiation was cost effective for targeted blood monitoring only in patients with HLA susceptibility alleles” [7].

To improve this idea, in this study, we aim to propose a new “HLA-guided treatment schedule”, whereby the HLA test is performed before the initiation of CLZ treatment in all patients, and if the TRS patients harbor “risk” HLA variants (HLA-B*59:01 for Japanese population and HLA-B 158T and HLA-DQB1 126Q for Caucasian population), we expect the psychiatrists to be aware of the specific risk for CIAG a priori resulting in preventing the CIAG onset, probably due to early “temporary cessation” of CLZ treatment to avoid the “complete discontinuation” of the CLZ treatment (e.g., cutoff definition for “temporary cessation”: ANC < 3000/mm^3^). Using this hypothesis, we addressed a question related to the cost-effectiveness of the HLA-guided treatment schedule compared with the “current treatment schedule” (i.e., “general treatment schedule without HLA test”), which involves blood monitoring without the HLA test. It is noteworthy that our study results mainly apply to subjects in Japan and the UK because (1) robust risk variants were detected in Japanese and Caucasian populations, and (2) an identical protocol was applied for the discontinuation of clozapine treatment based on the ANC cutoff (<1500/mm^3^). Furthermore, for comprehensive evaluation, we modeled several situations with multiple ANC thresholds (1000 mm^3^, 500/mm^3^, and 1500/mm^3^) for the CIAG definition to identify the best or estimate the next best option in actual clinical settings. This idea is derived from the presumption that the current ANC cutoff at 1500/mm^3^, which is being used in many countries (including Japan and the UK) but not in the United States of America (USA), is too conservative, and thus a better schedule can be proposed by this analysis.

## Methods

### Overview of the decision analytic model and “HLA-guided treatment schedule” (Fig. 1)

Cost-effectiveness analysis (CEA) followed the Guideline for Preparing Cost-Effectiveness Evaluation to the Central Social Insurance Medical Council [8] and Consolidated Health Economic Evaluation Reporting Standards (CHEERS) guideline [9].

**Fig. 1:**
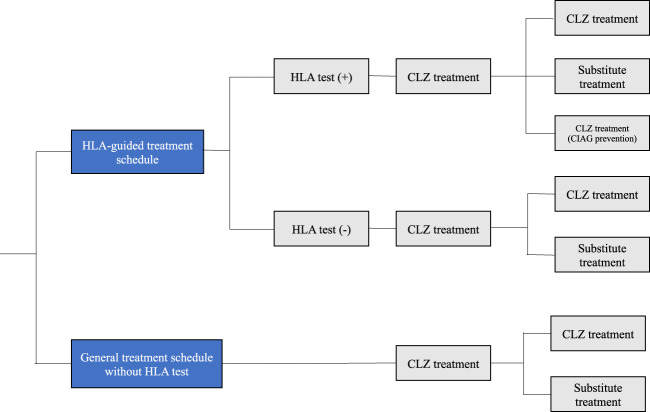
Decision tree schematic. The “general treatment schedule without HLA test” compared with the “HLA-guided treatment schedules”. CIAG clozapine-induced agranulocytosis/granulocytopenia.

In our decision analytic model, we simulated the cost-effectiveness using the hypothesis whereby the HLA test is performed for all subjects before the initiation of CLZ treatment: the risk alleles are HLA-B*59:01 [5] for Japanese population [frequency in dominant model (phenotype frequency) = HLA-B*59:01 ~4%], and HLA-B (158T)/DQB1 (126Q) in the Caucasian population [6] (phenotype frequency = “HLA-B 158T/HLA-DQB1 126Q” ~10.6%).

We modeled two alternative scenarios, as follows:

“HLA-guided treatment schedule”: In this scenario, we expect the situation where a priori information of the specific patients with risk HLA will alert the psychiatrists’ attitude, resulting in reduction of the overall CIAG onset rate. This is because the psychiatrists will be aware of the potential risk and will be sensitive to CIAG for these patients.

For this scenario, we created the term “CIAG prevention rate” (Supplementary Fig. 1). Using the reported clinical parameters [5, 6] and phenotype frequency, we (A) estimated the number of subjects with HLA variants who would develop CIA/CIG (Supplementary Fig. 1), (B) referenced the percentage of the admission rate of patients after the first 6 months of CLZ initiation (~60%) [10], and (C) simulated the percentage of subjects for whom CIAG would be prevented using the HLA test. Based on this assumption, we set the “CIAG prevention rate” at 30% for the base–case scenarios as an acceptable rate, based on previous findings [5, 6].

For example, in cases with ANC threshold at 1500/mm^3^, if we prevent all instances of CIG in subjects with HLA-B*59:01 or “HLA-B 158T/HLA-DQB1 126Q”, we can obtain a maximum CIAG prevention rate of ~70 and ~77% in Japan and in the UK, respectively using Bayes’ theorem (Supplementary Fig. 1). Therefore, if we prevent CIG in about 50% of such patients with risk of HLA variants, a “CIAG prevention rate” of ~30% can be obtained, we believe this is a realistic goal.

As a clinical predictor, we propose a clinical cutoff indicator for “temporary cessation” of CLZ in patients with high-risk HLA variants; the limitation in attaining this degree of “prevention rate” (~30%) is the fact that there is no difference in monitoring programs between the two alternatives presented in the model (“HLA-guided treatment schedule” and “General treatment schedule without HLA test,” mentioned below). Therefore, we consulted the CPMS of Japan and based on the data [the ANC trends in CIA (*N* = 69) and CIG (*N* = 48)], the onset of CIAG in these subjects was within 6 months of CLZ initiation), we estimated that an ANC cutoff of <3000/mm^3^ may be reasonable for predicting the development of CIAG; 84.1/85.4% of CIA/CIG decreased the minimum ANC at <3000/mm^3^ (Supplementary Fig. 2). Although the data did not take the risk of HLA variants into account, it is stressed that this cutoff was supported by the previous paper, where ANC at <3000/mm^3^ was the high risk of CIG [11]. We believe that this cutoff would enable careful monitoring of subjects with the risk of HLA variants.

In this scenario, if patients do not harbor the risk allele, we follow the “general treatment schedule without HLA test” in Japan or in the UK (see below).

“General treatment schedule without HLA test”: The comparison strategy is completely corresponding to or similar to the current monitoring schedule used in Japan or in the UK.

In either case, the monitoring schedule is identical to that in the “general treatment schedule without HLA test.” For the first 26 or 18 weeks of treatment in Japan and in the UK, respectively, weekly monitoring occurs and thereafter monitoring takes place every 2 weeks. However, if the WBC count or ANC decreases to <3000/mm^3^ or 1500/mm^3^, respectively, CLZ treatment should be discontinued (re-challenging for CIAG patients is also prohibited unless the CPMS committee gives permission based on the clinical course) at that moment.

In these models, we did not include the health state related to death and sepsis, because no case has been reported in such conditions after the CPMS started in Japan [12] or very low mortality (0.013%) based on the meta-analysis [13]. Moreover, we did not consider the possibility of CLZ discontinuation based on the WBC cutoff (<3000/mm^3^), because this “WBC count” definition was not a common reason for discontinuation (in such cases, the ANC usually decreased to 1500/mm^3^) [13].

### Population, model structure, and parameters

The target population was identical to that reported previously [7], i.e., adult men and women from Japan and the UK with TRS who are eligible for CLZ treatment. For the UK population, we targeted Caucasian-origin patients because higher risk of HLA variants was detected in the samples taken from Caucasian patients [HLA-B (158T)/DQB1 (126Q) [6]]. We used a Markov model for the transition probability (length: 1 month) to account for the trend of the susceptible timing of CIAG (Supplementary Table 1). The model incorporated the health states to reflect that patients were being administered either CLZ or substitute treatments. Patients who were being administered CLZ treatment were those with “Health state1”; they transitioned to “Health state2” (changing to “substitute treatment”) after the onset of CIAG as per the transition probabilities. In addition, we reflected these for calculation of the “CIAG onset” proportion into the health state (patients undergoing CLZ or substitute treatments) by adding the cost and utility (Supplementary Fig. 3).

We set the following parameters: (1) CIAG prevalence, (2) CIAG onset period, (3) allele frequency (AF) of risk HLA variants, (4) phenotype frequency (proportion of population homozygous or heterozygous for risk HLA variants) based on the Hardy–Weinberg equilibrium [1-(1-AF) [2]], and (5) positive predictive value (PPV) of the risk HLA variants based on the results presented in the previous papers [5, 6] (Supplementary Table 2).

The outcome included the mean cost-per-patient and QALYs-per-patient for the calculation of the ICER over a 10-year time period. In particular, in Japan, the mean age of the patients at the start of CLZ treatment was 40.6 years [12], and antipsychotics were recommended to be continued. Thus, we assumed that patients with TRS receive CLZ treatment for at least 10 years, if tolerated. However, for comprehensive evaluation, we included additional analysis for various time-periods (from 3 to 20 years).

### Cost estimates and utilities (Supplementary Table 3)

Costs related to medical fees were calculated as per the direct Medical Care Expenditure based on the “Medical-fee point” system in Japan and the National Health Service in the UK (as on 1/Apr/2020) [14, 15]. All costs were converted to Great Britain Pounds (GBP, £) at a conversion rate of 132.8 Japanese Yen (JPY) to £1 (as on 1/Apr/2020).

The major costs incurred were as follows:

1. Cost of treatment for CIAG: £985.8 and £469.48 for the Japanese and the UK [15] populations, respectively.
2. Cost of CLZ/day—calculated as per the pharmaceutical price of CLZ (£2.37/100 mg) and daily mean dosage of CLZ186.41 mg [12]: £4.42 for the Japanese population. CLZ (£0.41/100 mg) and daily mean dosage of CLZ 300 mg: £1.23 for the UK population [14, 16].
3. Cost of substitute treatment/day—two types of second-generation antipsychotics are commonly used for TRS in Japan; hence, we calculated this fee as follows: cost × percentage of the first-line drugs used in Japan (risperidone: 30%, aripiprazole: 18.7%, olanzapine: 18.1%, and pariperidone: 8.4%): £7.52 [17]: one type of second-generation antipsychotic is commonly used for schizophrenia calculated this fee by weighting the cost × the percentage of the first-line drugs used in the UK (risperidone: 21.5%, aripiprazole: 10.8%, olanzapine: 19.7%, quetiapine: 42.8%, and amisulpride: 5.2%): £5.11 [14, 18].

We used the “utility” based on the data from the previous paper that analyzed the CEA of CLZ treatment based on HLA variants for the Caucasian popul,ation [7] because there were no data from Japan, the UK, or other countries that would enable the estimation of the precise utility for TRS patients, who did and did not undergo CLZ treatment. The utility for patients undergoing CLZ treatment was set at 0.693 [7] and that for patients undergoing substitute treatment was set at 0.560 [7]. The utility derived from CIAG was not accounted for in this analysis, because the mortality rate was extremely low, and the median treatment duration of CIAG was 4.5 days as observed in Japan [12].

### Incremental cost-effectiveness ratio

We obtained all the costs and QALYs for the “HLA-guided treatment schedule” and “general treatment schedule without HLA test” and calculated the ICER using the following formula:

$$\frac{{{\rm{Cost}}\left( {{\rm{HLA}} - {\rm{guided}}\,{\rm{treatment}}\,{\rm{schedule}}} \right) - {\rm{Cost}}\,({\rm{general}}\,{\rm{treatment}}\,{\rm{schedule}}\,{\rm{without}}\,{\rm{HLA}}\,{\rm{test}})}}{{{\rm{QALY}}({\rm{HLA}} - {\rm{guided}}\,{\rm{treatment}}\,{\rm{schedule}}) - {\rm{QALY}}\,({\rm{general}}\,{\rm{treatment}}\,{\rm{schedule}}\,{\rm{without}}\,{\rm{HLA}}\,{\rm{test}})}}$$

Discount rates of 2% (for Japanese patients) or 3.5% (for the UK patients) were applied to the costs and QALYs as per the above guideline. The cost-per-QALY thresholds were set at <£37,650.6 (5,000,000 JPY) for Japanese patients and <£30,000 for UK patients), as recommended in the Japanese and UK guidelines [8].

### Base–case scenario analysis

The viewpoint was set from a healthcare provider’s perspective in the CEA. The primary base–case analysis is to compare between “HLA-guided treatment schedule” and “general treatment schedule without HLA test” with the ANC cutoff set at 1500/mm^3^, which is completely corresponding to that used in Japan and UK.

### Explorative analysis

As part of the secondary analysis, we explored the cost-effectiveness in various scenarios with multiple ANC thresholds (500/mm^3^, 1000/mm^3^, and 1500/mm^3^|with/without HLA test) under the same parameters used in the base–case scenario analysis. However, most of the schedules revealed worse cost as well as QALYs compared with the “general treatment schedule without HLA test” with ANC thresholds at 500/mm^3^. This indicates CEA is not appropriate, because obvious better outcome would be obtained from this schedule, as discussed below. Whereas, we plotted the cost/QALYs relationships for each schedule (cost-effectiveness graph) to visualize which is the better one compared to the “current schedule,” because psychiatrists tend to select candidate “next treatment option” as safety consideration (i.e., this provides important information for selecting gradual shift for better treatment option in the real-clinical setting).

### Sensitivity analyses

For the base–case scenario, firstly we conducted one-way sensitivity analysis by varying the major parameters in the model within appropriate reliability values or confidence intervals (CIs) to evaluate the robustness of the results. We varied the following six parameters: (1) the “CIAG prevention rate”, (2) PPV of the HLA test, (3) cost of CIAG treatment, (4) daily treatment cost of CLZ, (5) daily cost of substitute treatment, and (6) discount rate. However, these parameters are not perfect estimates, specifically for the “CIAG prevention rate” and PPV of the HLA. Thus, we emphasized on the probabilistic sensitivity analysis conducted using a Monte Carlo simulation by varying each parameter (95% CIs or clinically reasonable ranges). We set the number of simulations to 100,000, based on randomly assigned parameters and obtained 95% CIs for the cost and QALY values (i.e., 0.025–0.975 percentile).

For the explorative analysis to overview the best (or better) scenarios, we only included one-way sensitivity analysis for “CIAG prevention rate” in “HLA-guided treatment schedule” ranging 20–80%.

TreeAgePro software (2019 version, TreeAge Software Inc., MA, USA) was used to create the model for calculating cost-effectiveness and sensitivity with these analyses.

## Results

### Base–case scenario analysis

In the primary base–case analysis (“HLA-guided treatment schedule” vs. “general treatment schedule without HLA test”: ANC threshold at 1500/mm^3^) for the “point estimation”, the total cost for a 10-year duration under the “HLA-guided treatment schedule” was estimated to be £16,552/£4281 (Japan/UK), whereas that of the “general treatment schedule without HLA test” was estimated at £16,487/£4211 (Japan/UK), representing an increase of £65/£70 (Japan/UK). The average QALYs under the “HLA-guided treatment schedule” was 6.22917/5.82990 (Japan/UK), while that under the “general treatment schedule without HLA test” was 6.22608/5.82665 (Japan/UK), indicating a gain of 0.00309/0.00326 QALYs (Japan/UK). Based on these findings, the ICERs for the “HLA-guided treatment schedule” was £21,024/£21,343 (Japan/UK) per QALY for a 10-year duration; thus, this ICER was lower than the cost-per-QALY threshold of £37,650.6 (5,000,000 JPY) and £30,000 (Table 1).

**Table 1:** Base–case scenario results.

	General treatment schedule without HLA test	HLA-guided treatment schedule
Cost per patient/10 years (JPN/UK)	£16,487/£4211	£16,552/£4281
Incremental Cost (JPN/UK)		£65/£70
Effect per patient/10 years (QALYs: JPN/UK)	6.22608/5.82665	6.22917/5.82990
Incremental effect (QALYs: JPN/UK)		0.00309/0.00326
ICER (JPN/UK)		£21,024/£21,343

*JPN* Japan, *UK* United Kingdom, *QALY* quality-adjusted life years, *ICER* incremental cost-effectiveness ratio.

**Fig. 2:**
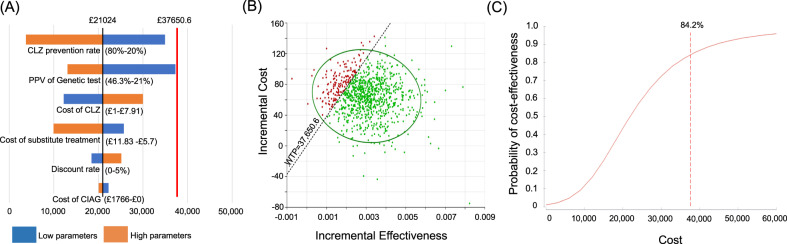
Base–case scenario analysis in Japan. **A** Tornado plot of the one-way sensitivity analysis: The black vertical line indicates the incremental cost-effectiveness ratio (ICER) of the base-case analysis. The red vertical line indicates the cost-per QALY threshold. The numbers are the ranges used in the sensitivity analysis. The blue shaded bars represent lower parameters than those used in the base–case analysis, and the red shaded bars represent higher parameters than those used in the base-case analysis. **B** Scatter plot for incremental cost and effectiveness: green dots indicate ICERs within willing to pay threshold and red dots indicate out of the threshold. **C** Cost-effectiveness acceptability curve, CLZ clozapine, PPV positive predictive value, CIAG clozapine-induced agranulocytosis/granulocytopenia, WTP willing to pay.

**Fig. 3:**
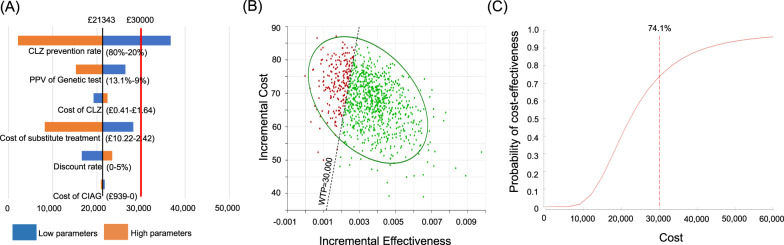
Base–case scenario analysis in the UK. **A** Tornado plot of the one-way sensitivity analysis: the black vertical line indicates the incremental cost-effectiveness ratio (ICER) of the base-case analysis. The red vertical line indicates the cost-per QALY threshold. The numbers are the ranges used in the sensitivity analysis. The blue shaded bars represent lower parameters than those used in the base–case analysis, and the red shaded bars represent higher parameters than those used in the base–case analysis. **B** Scatter plot for incremental cost and effectiveness: green dots indicate ICERs within willing to pay threshold and red dots indicate out of the threshold. **C** Cost-effectiveness acceptability curve CLZ clozapine, PPV positive predictive value, CIAG clozapine-induced agranulocytosis/granulocytopenia, WTP willing to pay.

The results of the one-way sensitivity analysis for the primary analysis, involving the six major model parameters, to assess the robustness of the model are shown in Figs. 2 and 3 (A) and Supplementary Tables 4 and 5. Most of the parameters did not dramatically influence the ICER. In fact, the estimated major factor was the “CIAG prevention rate” specifically in the UK; for UK samples, less than 23.4% of the “CIAG prevention rate” surpassed the cost-per QALY threshold.

Next, we conducted a probabilistic sensitivity analysis and obtained a 95% CI for the cost and QALYs, based on 100,000 simulations. The probabilistic estimate of the total cost under the “HLA-guided treatment schedule” was £16,551/£4279 (Japan/UK) and that for QALYs was 6.22966/5.82992 (Japan/UK: Supplementary Table 6). In contrast, the estimates of “general treatment schedule without HLA test” showed that the total cost was £16,487/£4210 (Japan/UK) and that for QALYs was 6.22657/5.82667 (Japan/UK: Supplementary Table 6). Consequently, the average ICER was £20,995/£21,373 (Japan/UK) and the probability (willingness to pay) was 84.2%/74.1% (Japan/UK) against the cost-per-QALY threshold, indicating the “HLA-guided treatment schedule” showed the desirable range of ICER, shown in Figs. 2 and 3B, C and Supplementary Table 6.

Finally, we checked the relationships between the ICERs and time durations by changing the values (3–20 years) to secure our setting at 10 years as reasonable. In this simulation, we found higher ICERs over the cost-per-QALY threshold (£37,650.6 and £30,000, for Japanese and the UK patients, respectively) at time-horizons of 3-6/3-7 (Japan/UK) years; however, at ≥7/8 (Japan/UK) years, the ICERs were below the threshold (Supplementary Fig. 4 and Supplementary Tables 7 and 8).

### Explorative analysis: the better or the best schedule in terms of cost-effectiveness

In either analysis for the Japanese and the UK populations, all of the treatment schedules were more effective and less costly compared to the “general treatment schedule without HLA test” with ANC threshold at 1500/mm^3^ (Table 2). It implied that this “general treatment schedule without HLA test” with ANC threshold at 1500/mm^3^ (current schedule used in Japan and UK) was always worse in terms of cost-effectiveness than “HLA-guided treatment schedules” with ANC thresholds at 1000/mm^3^ and 500/mm^3^.

**Table 2:** Costs and effects of changing the ANC cutoff line (500/mm^3^–1000/mm^3^–1500/mm^3^) in Japan and the UK.

Cutoff line	Cost/QALYs	JPN		UK	
		HLA-guided treatment schedule	general treatment schedule without HLA test	HLA-guided treatment schedule	general treatment schedule without HLA test
500/mm^3^	Cost per patient £	16,257 (16,255–16,258)	16,173	4021 (4008–4023)	3,928
	QALYs for ten years	6.27343 (6.27389–6.27334)	6.27316	5.85539 (5.85672–5.85512)	5.85459
	ICER £/ (QALY): HLA-guided treatment schedule vs. general treatment schedule without HLA test	307,957 (111,753–464,946)		116,190 (37,712–178,986)	
1000/mm^3^	Cost per patient £	16,401 (16,377–16,405)	16,329	4151 (4119–4157)	4070
	QALYs for ten years	6.25207 (6.25607–6.25126)	6.24967	5.84267 (5.84698–5.84198)	5.84062
	ICER £/ (QALY): HLA-guided treatment schedule vs. general treatment schedule without HLA test	29,836 (7444–47,810)		39,512 (8958–63,968)	
1500/mm^3^	Cost per patient £	16,552 (16,517–16,558)	16,487	4281 (4230–4291)	4211
	QALYs for 10 years	6.22917 (6.23434–6.22813)	6.22608	5.8299 (5.83533–5.82882)	5.82665
	ICER £/ (QALY): HLA-guided treatment schedule vs. general treatment schedule without HLA test	21,024 (3729–34,920)		21,343 (2145–36,715)	

The numbers in the parentheses indicates results from the sensitivity analysis for CIAG prevention rate (80–20%).

*JPN* Japan, *UK* United Kingdom, *ICER* incremental cost-effectiveness ratio, *QALY* quality-adjusted life year, *CIAG* Clozapine-induced agranylocytosis/granulocytopenia.

Also, based on the cost-effectiveness graph, it clearly indicated that the best schedule was “general treatment schedule without HLA test” with ANC threshold at 500/mm^3^ (Table 2), which is completely corresponding the current schedule used in the USA. In this case, it is obvious that the CEA is not appropriate in the comparison between this schedule and any “HLA-guided treatment schedule”, because this “general treatment schedule without HLA test (ANC threshold at 500/mm^3^)” always showed better cost as well as QALYs, indicating no ICER was calculated.

## Discussion

In this study, we proposed a novel clinically matched schedule for treating TRS patients with CLZ, an “HLA-guided treatment schedule”, and found it to be more cost effective than the “general treatment schedule without HLA test” (i.e., the current treatment schedule) used in Japan and the UK (ANC cutoff at <1500/mm^3^). In addition, we simulated the cost-effectiveness at various ANC cutoffs to define CIG, and the “general treatment schedule without HLA test (ANC < 1500/mm^3^)” is not the best choice for the CLZ treatment in Japan and the UK; it is too conservative from viewpoints of cost-effectiveness.

The “HLA-guided treatment schedule” aims to prevent CIAG by identifying patients with the high risk of HLA variants a priori, which is specifically effective for Japanese and Caucasian subjects in the UK and many other countries except the USA. This is because ANC thresholds for CIAG are still set at a stringent level, such as 1500/mm^3^, indicating that CIG can be prevented in many subjects. For the remaining patients who did not have the risk, this scenario is also acceptable for psychiatrists, as psychiatrists should monitor the ANC in an identical manner, which is done regularly for all patients under the CPMS systems.

Compared with the scenario or the proposed schedule in this study, the scenario of the previous CEA (without considering the HLA test) in the USA [19] was slightly extreme, indicating that the current blood monitoring scheme was not as cost effective as the “no blood monitoring” schedule, mainly owing to the limited difference in the mortality attributed to CIAG. In another CEA (also for patients in the USA) that used HLA variants [7], their “HLA-guided treatment schedule” was a cost-effective option, consistent with our results, although monitoring was performed only for those with the risk variants; patients without the risk variant were not monitored at all. However, in the actual clinical setting, even if the CEA shows that the “no blood monitoring” or “blood monitoring only for targeted patients” schedules are cost effective, we believe that this is not realistic from the psychological viewpoint of the psychiatrists and patients.

The sensitivity analyses support our base-case “point-estimate” findings as acceptable; they indicate that the “PPV” was the most sensitive to the ICER in Japan; however, it was within the cost-QALY threshold. Other factors also do not largely impact ICER, and specifically, it is noteworthy that “CIAG prevention rate” (even at 20%) introduces cost-effectiveness to the HLA-guided treatment schedule in Japan. In fact, we estimated and set this rate at 30% in the base–case analysis, wherein 30% of the cases of CIAG could be prevented, and we believe that this estimate is realistic. In contrast, CEA in the UK revealed that “CIAG prevention rate” was the major factor (23.4% was the cutoff that surpass the cost-per QALY threshold). Although this did not exert a strong influence on our results, we can preclude that this treatment schedule is more applicable in Japan under the situation, where we used uncertain parameters.

Nevertheless, based on the results wherein the “HLA-guided treatment schedule” has proven more cost effective in many countries applying stringent CPMS protocol (except for patients in the USA), we believe that it is important to increase the “absolute number” of CLZ treatments for TRS in the future; more patients should be prescribed CLZ to improve the QOL with a reasonable increase in the cost. In other words, CLZ treatment is able to enhance the patient’s QOL at a lower cost. Further, less anxiety about CIAG (a type of “intangible benefit”) would improve the effect on patients with TRS, who have not currently been prescribed CLZ.

Thus, the best way to increase the “absolute number” of CLZ treatments is to apply a relaxed definition of CIAG; in fact, there is no robust evidence in favor of the application of this cutoff (ANC at 1500/mm^3^). Our explorative analysis supported this and revealed that the best scenario was “general treatment schedule without HLA test” with ANC threshold at 500/mm^3^. This is completely corresponding to the CPMS schedule currently applied in the USA. This is derived from the followings: (1) all of the CIG subjects were not discontinued (thereby increasing the QALYs) and (2) there was no cost for HLA typing. From viewpoint of CEA, we can conclude this schedule is the most optimal protocol; however, due to the psychiatrists’ attitude for safety consideration for patients, drastic change of protocol from the stringent/safety protocol (e.g., ANC 1500/mm^3^) to such a little bit “too relaxed” protocol is not corresponded to the needs in the clinical setting. Therefore, our explorative analysis indicated the clinically acceptable “gradual shift” of the protocol; “HLA-guided treatment schedule” with ANC threshold at 1000/mm^3^ may be a candidate option, because currently used option (corresponding to “general treatment schedule without HLA test” with ANC threshold at 1500/mm^3^) was even worse than this HLA-guided schedule with ANC threshold at 1000/mm^3^. Therefore, we have revealed possible clinical indicator, which is the next protocol, to apply the CLZ treatment to reduce cost and increase QALY with acceptable clinical sense of psychiatrists.

Our study has certain limitations. First, the “prevention rate” applied in this study (30%) has not been validated. Although we consider that this estimate is not too optimistic, prospective studies on this subject are warranted. Second, we showed the ICERs for patients in Japan and the UK; however, the results should be interpreted carefully in the context of populations from other countries. We believe that not enough attention has been given to the differences in costs and outcomes between the Japanese and UK analyses. Clinical differences related to relevant HLA and the pool of antipsychotics have been considered; however, it does not appear that there are any differences accounted for regarding how health systems may approach treatment of CIAG. Third, we did not consider other adverse effects associated with the alternative treatments in this model; however, the main adverse effect of second-generation antipsychotics is metabolic syndrome, which is also an adverse effect of CLZ. Fourth, our results can only be applied to Japanese and Caucasian populations. Further PGx studies and CEA analysis on different populations are required due to differences like the Duffy-null genotype, which is associated with decreased ANC in African population [20].

In conclusion, this study showed that the “HLA-guided treatment schedule” with CLZ treatment for TRS harboring high risk HLA may be clinically reasonable and cost-effective compared to the protocol currently used in Japan and the UK. Preventing CIAG and maintaining CLZ treatment contributes to a higher number of QALYs, thus lowering the ICER with the HLA test. Also, our finding provided a key to select better protocol based on cost-effectiveness, for moving next step to select “better” treatment schedule. Although further prospective/observational studies to establish definitive parameters are essential, we believe that the evidence from the present study provides new ideas for optimizing the clinical guidelines of CLZ treatment in TRS patients. In addition, being aware of personal “risk HLA” or variants related to PGx traits is important for the prevention of serious adverse effects; every person should notice their potential PGx risk a priori, not only with respect to CLZ treatment, opening gate to a new era of personalized medicine or more broadly precision medicine. To do so, obviously, lowering genotype cost is essential.

## Supplementary information

Supplementary Tables

Supplementary Figures
