# Supplementary material for: Cost effectiveness of pharmacogenetic-guided clozapine administration based on risk of HLA variants in Japan and the UK
Source: Transl Psychiatry. 2021 Jul 7;11:362. doi: 10.1038/s41398-021-01487-4 (PMC8260588; doi:10.1038/s41398-021-01487-4)
Supplement: Supplementary file 1 — Supplementary Tables [file 41398_2021_1487_MOESM1_ESM.pdf]

		HLA-guided treatment schedule		general treatment schedule without HLA test
CIAG transition probability	For Japan	HLA-B*59:01 (+)	HLA-B*59:01 (-)	All patients
	1-4 months	0.0399	0.00657	0.00835
	5-12 months	0.0117	0.00167	0.00213
	13-120 months	0.000448	4.16E-05	5.32E-05
	For the UK	HLA-B 158T/HLA-DQB1 126Q (+)	HLA-B 158T/HLA-DQB1 126Q (-)	All patients
	1-4 months	0.0241	0.00592	0.00783
	6-120 months	0.000103	2.39E-05	3.181E-05

Supplementary Table1: CIAG transition probability associated with health states 1 and 2

CIAG: Clozapine-induced agranylocytosis/granulocytopenia

CIAG transition probability for the Japanese population was estimated based on the trend of the susceptible timing of CIAG (start to 4 months: 60%, 6–12 months: 30%, at 13 months: 10%, Inada et al., 2018)

CIAG transition probability for the UK population was estimated based on the trend of the susceptible timing of CIAG (start to 6 months: 90%, 6 months: 10%)

Parameter	JPN/UK	Mean	Lowest value for sensitivity analysis	Highest value for sensitivity analysis	Probabilistic sensitivity analysis	Type of distribution	Distribution Parameter	References
CIAG prevalence	JPN	5.44%			NO			12
	UK	3.43%			NO			11
allele frequency of risk HLA variants	JPN	2%			NO			HLA Laboratory*
	UK	5.46%			NO			6
phenotype frequency	JPN	3.96%			NO			
	UK	10.60%			NO			
PPV of HLA test	JPN	32.30%	21%	46.3%	YES	Beta	alpha:117.2 beta:236.4	5
	UK	10.60%	9%	13.1%	YES	Beta	alpha:98.6 beta:840.2	6

Supplementary Table2: Input parameters

CIAG: Clozapine-induced agranylocytosis/granulocytopenia, JPN: Japan, UK: United Kingdom, PPV: positive predictive value

CIAG onset period are listed in Supplementary Table1.

*: http://hla.or.jp/med/frequency_search/en/haplo/

Parameter	JPN/UK	Mean	Lowest value for sensitivity analysis	Highest value for sensitivity analysis	Probabilistic sensitivity analysis	Type of distribution	Distribution Parameter	References
Cost of CIAG £	JPN	985.8	0	1766	YES	Gamma	alpha:1.48 lambda:1.5E-3	12
	UK	469.48	0	939	YES	Gamma	alpha:4 lambda:8.5E-3	15
Cost of CLZ/day £	JPN	4.42	1	7.91	YES	Gamma	alpha:6.05 lambda:1.37	12
	UK	1.23	0.41	1.64	YES	Gamma	alpha:37.8 lambda:30.75	14,16
Cost of substitute/day £	JPN	7.52	5.7	11.83	YES	Gamma	alpha:9.92 lambda:1.32	17
	UK	5.11	2.42	10.22	YES	Gamma	alpha:104.4 lambda:20.44	14,16
Cost of HLA test £	JPN	86.6			NO			
	UK	100			NO			
Cost of regular blood test*/month £	JPN	30.27			NO			
	UK	10.6			NO			
Utility for patients undergoing clozapine treatment	JPN/UK	0.693			YES	Beta	alpha:575 beta:255	7
Utility for patients undergoing substitute treatment	JPN/UK	0.560			YES	Beta	alpha:86 beta:67	7
CIAG prevention rate	JPN/UK	30%	20%	80%	YES	Beta	alpha:24.9 beta:58.1	

Supplementary Table3: Cost estimates, utilities, and other parameter used in the sensitivity analysis

CIAG: Clozapine-induced agranylocytosis/granulocytopenia, CLZ: clozapine, JPN: Japan, UK: United Kingdom, G-CSF: granulocyte colony stimulating factor

*Cost for the regular blood examination for patients who would be given clozapine treatment. If patients developed CIAG, this fee would not be counted.

		general treatment schedule without HLA test	HLA-guided treatment schedule
CLZ prevention rate (0.2-0.8)	Cost per patient/10 years £	16,486	16,558-16,517
	Incremental Cost £		72-31
	Effect per patient /10 years (QALYs)	6.22608	6.22813-6.23434
	Incremental Effect (QALYs)		0.00206-0.00826
	ICER £/ (QALY)		34,920-3,729
PPV of Genetic test (0.21-0.463)	Cost per patient/10 years £	16,486	16,560-16,543
	Incremental Cost £		73-56
	Effect per patient /10 years (QALYs)	6.22608	6.22803-6.23040
	Incremental Effect (QALYs)		0.00196-0.00432
	ICER £/ (QALY)		37,261-13,052
Cost of CLZ/day £ (1-7.91)	Cost per patient/10 years £	6,578-26,597	6,617-26,689
	Incremental Cost £		37-93
	Effect per patient /10 years (QALYs)	6.22608	6.22917

	Incremental Effect (QALYs)		0.00309
	ICER £/ (QALY)		12,230-29,997
Cost of substitute/day £ (5.7-11.83)	Cost per patient/10 years £	16,212-17,139	16,291-17,170
	Incremental Cost £		79-31
	Effect per patient /10 years (QALYs)	6.22608	6.23020
	Incremental Effect (QALYs)		0.00309
	ICER £/ (QALY)		25,695-9934
Cost of CIAG £ (0-1766)	Cost per patient/10 years £	16,434-16,529	16,503-16,591
	Incremental Cost £		69-61
	Effect per patient /10 years (QALYs)	6.22608	6.23123
	Incremental Effect (QALYs)		0.00309
	ICER £/ (QALY)		22,249-20,054
Discount rate (0-0.05)	Cost per patient/10 years £	18,163-14,393	18,226-14,460
	Incremental Cost £		63-68
	Effect per patient /10 years (QALYs)	6.86254-5.43114	6.86710-5.43473
	Incremental Effect (QALYs)		0.00341-0.00269
	ICER £/QALYs		18,440-25,125

Supplementary Table4: One-way sensitivity analysis for the Japanese population

CLZ: clozapine, ICER: incremental cost-effectiveness ratio, QALY: quality-adjusted life year

		general treatment schedule without HLA test	HLA-guided treatment schedule
CLZ prevention rate (0.2-0.8)	Cost per patient/10 years £	4,211	4,291-4,230
	Incremental Cost £		80-19
	Effect per patient /10 years (QALYs)	5.82665	5.82882 -5.83533
	Incremental Effect (QALYs)		0.00217 -0.00869
	ICER £/ (QALY)		36,715-2,145
PPV of Genetic test (0.09-0.131)	Cost per patient/10 years £	4,211	4,285-4,273
	Incremental Cost £		72-59
	Effect per patient /10 years (QALYs)	5.82665	5.82944 -5.83071
	Incremental Effect (QALYs)		0.00279 -0.00406
	ICER £/ (QALY)		26,456 -15,256
Cost of CLZ/day £ (0.41-1.64)	Cost per patient/10 years £	1,954-5,340	2,016-5,413
	Incremental Cost £		63 -73
	Effect per patient /10 years (QALYs)	5.82665	5.8299
	Incremental Effect (QALYs)		0.00326
	ICER £/ (QALY)		19,235-22,397
Cost of substitute/day £ (2.42-10.22)	Cost per patient/10 years £	3,968-4,672	4,060-4,699
	Incremental Cost £		92-27

	Effect per patient /10 years (QALYs)	5.82665	5.8299
	Incremental Effect (QALYs)		0.00326
	ICER £/ (QALY)		28,260-8,205
Cost of CIAG £ (0-939)	Cost per patient/10 years £	4,195-4,227	4,266-4,295
	Incremental Cost £		71-68
	Effect per patient /10 years (QALYs)	5.82665	5.8299
	Incremental Effect (QALYs)		0.00326
	ICER £/ (QALY)		21,774-20,912
Discount rate (0-0.05)	Cost per patient/10 years £	4,962-3,943	5,026-4,015
	Incremental Cost £		64-71
	Effect per patient /10 years (QALYs)	6.88596 -5.44930	6.88982 -5.45234
	Incremental Effect (QALYs)		0.00386 -0.00304
	ICER £/QALYs		16,594-23,491

Supplementary Table5: One-way sensitivity analysis for the UK population

CLZ: clozapine, ICER: incremental cost-effectiveness ratio, QALY: quality-adjusted life year

	JPN/UK	HLA-guided treatment schedule (95%CI)	general treatment schedule without HLA test (95%CI)
Cost per patient £	JPN	16,551 (8,366-28,559)	16,487 (8,321-28,437)
	UK	4,279 (3,276-5,451)	4,210 (3,209-5,378)
QALYs for ten years	JPN	6.22966 (5.95397-6.49825)	6.22657 (5.95143-6.49488)
	UK	5.82992 (5.56793-6.08483)	5.82667 (5.56492-6.08109)

Supplementary Table6: Result of probabilistic sensitivity analysis based on 100,000 simulations

QALY: quality-adjusted life year, CI: confidence interval, JPN: Japan, UK: United Kingdom, ICER: incremental cost-effectiveness ratio

	general treatment schedule without HLA test	HLA-guided treatment schedule		general treatment schedule without HLA test	HLA-guided treatment schedule
Cost per patient/3 years	5,364	5,443	Cost per patient/12 years £	19,395	19,456
Incremental Cost £		78	Incremental Cost £		61
Effect per patient /3 years (QALYs)	2.00203	2.00108	Effect per patient /12 years (QALYs)	7.33256	7.32891
Incremental Effect (QALYs)		0.00094	Incremental Effect (QALYs)		0.00365
ICER £/ (QALY)		83,089	ICER £/ (QALY)		16,828
Cost per patient/4 years £	7,048	7,124	Cost per patient/13 years £	20,807	20,867
Incremental Cost £		76	Incremental Cost £		60
Effect per patient /4 years (QALYs)	2.64258	2.64131	Effect per patient /13 years (QALYs)	7.86799	7.86407
Incremental Effect (QALYs)		0.00127	Incremental Effect (QALYs)		0.00393
ICER £/ (QALY)		60,181	ICER £/ (QALY)		15,222
Cost per patient/5 years £	8,699	8,773	Cost per patient/14 years £	22,191	22,249
Incremental Cost £		74	Incremental Cost £		58
Effect per patient /5 years (QALYs)	3.27050	3.26891	Effect per patient /14 years (QALYs)	8.39287	8.38867
Incremental Effect (QALYs)		0.00159	Incremental Effect (QALYs)		0.00420
ICER £/ (QALY)		46,841	ICER £/ (QALY)		13,848
Cost per patient/6 years £	10,318	10,390	Cost per patient/15 years £	23,549	23,606
Incremental Cost £		72	Incremental Cost £		56

Effect per patient /6 years (QALYs)	3.88604	3.88414	Effect per patient /15 years (QALYs)	8.90739	8.90293
Incremental Effect (QALYs)		0.00190	Incremental Effect (QALYs)		0.00446
ICER £/ (QALY)		38,111	ICER £/ (QALY)		12,660
Cost per patient/7 years £	11,906	11,976	Cost per patient/16 years £	24,881	24,936
Incremental Cost £		71	Incremental Cost £		55
Effect per patient /7 years (QALYs)	4.48943	4.48723	Effect per patient /16 years (QALYs)	9.41177	9.40705
Incremental Effect (QALYs)		0.00221	Incremental Effect (QALYs)		0.00472
ICER £/ (QALY)		31,952	ICER £/ (QALY)		11,623
Cost per patient/8 years £	13,463	13,531	Cost per patient/17 years £	26,186	26,240
Incremental Cost £		69	Incremental Cost £		53
Effect per patient /8 years (QALYs)	5.08093	5.07843	Effect per patient /17 years (QALYs)	9.90620	9.90122
Incremental Effect (QALYs)		0.00251	Incremental Effect (QALYs)		0.00498
ICER £/ (QALY)		27,374	ICER £/ (QALY)		10,708
Cost per patient/9 years £	14,989	15,056	Cost per patient/18 years £	27,467	27,519
Incremental Cost £		67	Incremental Cost £		52
Effect per patient /9 years (QALYs)	5.66077	5.65797	Effect per patient /18 years (QALYs)	10.39088	10.38566
Incremental Effect (QALYs)		0.00280	Incremental Effect (QALYs)		0.00523
ICER £/ (QALY)		23,838	ICER £/ (QALY)		9,897
Cost per patient/10 years £	16,487	16,552	Cost per patient/19 years £	28,723	28,773
Incremental Cost £		65	Incremental Cost £		50

Effect per patient /10 years (QALYs)	6.22917	6.22608	Effect per patient /19 years (QALYs)	10.86601	10.86053
Incremental Effect (QALYs)		0.00309	Incremental Effect (QALYs)		0.00547
ICER £/ (QALY)		21,024	ICER £/ (QALY)		9,171
Cost per patient/11 years £	17,955	18,018	Cost per patient/20 years £	29,954	30,003
Incremental Cost £		63	Incremental Cost £		49
Effect per patient /11 years (QALYs)	6.78636	6.78298	Effect per patient /20 years (QALYs)	11.33176	11.32605
Incremental Effect (QALYs)		0.00338	Incremental Effect (QALYs)		0.00571
ICER £/ (QALY)		18,732	ICER £/ (QALY)		8,519

Supplementary Table7: Incremental cost-effectiveness ratios (ICERs) in different time horizon years for Japanese population

ICER: incremental cost-effectiveness ratio, QALY: quality-adjusted life year

	general treatment schedule without HLA test	HLA-guided treatment schedule		general treatment schedule without HLA test	HLA-guided treatment schedule
Cost per patient/3 years £	1,478	1,567	Cost per patient/12 years £	4,881	4,945
Incremental Cost £		89	Incremental Cost £		65
Effect per patient /3 years (QALYs)	1.96372	1.96477	Effect per patient /12 years (QALYs)	6.76971	6.77351
Incremental Effect (QALYs)		0.00105	Incremental Effect (QALYs)		0.00379
ICER £/ (QALY)		84,824	ICER £/ (QALY)		17,040
Cost per patient/4 years £	1,908	1,995	Cost per patient/13 years £	5,199	5,261
Incremental Cost £		86	Incremental Cost £		62
Effect per patient /4 years (QALYs)	2.57424	2.57564	Effect per patient /13 years (QALYs)	7.21747	7.22152
Incremental Effect (QALYs)		0.00140	Incremental Effect (QALYs)		0.00405
ICER £/ (QALY)		61,515	ICER £/ (QALY)		15,397
Cost per patient/5 years £	2,325	2,408	Cost per patient/14 years £	5,506	5,566
Incremental Cost £		83	Incremental Cost £		60
Effect per patient /5 years (QALYs)	3.16407	3.16581	Effect per patient /14 years (QALYs)	7.65008	7.65437
Incremental Effect (QALYs)		0.00174	Incremental Effect (QALYs)		0.00430
ICER £/ (QALY)		47,866	ICER £/ (QALY)		13,995
Cost per patient/6 years £	2,728	2,808	Cost per patient/15 years £	5,803	5,861

Incremental Cost £		80	Incremental Cost £		58
Effect per patient /6 years (QALYs)	3.73392	3.73598	Effect per patient /15 years (QALYs)	8.06806	8.07259
Incremental Effect (QALYs)		0.00206	Incremental Effect (QALYs)		0.00454
ICER £/ (QALY)		38,906	ICER £/ (QALY)		12,786
Cost per patient/7 years £	3,117	3,195	Cost per patient/16 years £	6,089	6,145
Incremental Cost £		77	Incremental Cost £		56
Effect per patient /7 years (QALYs)	4.28445	4.28683	Effect per patient /16 years (QALYs)	8.47191	8.47667
Incremental Effect (QALYs)		0.00238	Incremental Effect (QALYs)		0.00477
ICER £/ (QALY)		32,577	ICER £/ (QALY)		11,732
Cost per patient/8 years £	3,494	3,569	Cost per patient/17 years £	6,366	6,420
Incremental Cost £		75	Incremental Cost £		54
Effect per patient /8 years (QALYs)	4.81634	4.81902	Effect per patient /17 years (QALYs)	8.86210	8.86709
Incremental Effect (QALYs)		0.00268	Incremental Effect (QALYs)		0.00499
ICER £/ (QALY)		27,870	ICER £/ (QALY)		10,807
Cost per patient/9 years £	3,859	3,931	Cost per patient/18 years £	6,634	6,686
Incremental Cost £		72	Incremental Cost £		52
Effect per patient /9 years (QALYs)	5.33020	5.33317	Effect per patient /18 years (QALYs)	9.23909	9.24430
Incremental Effect (QALYs)		0.00297	Incremental Effect (QALYs)		0.00520
ICER £/ (QALY)		24,234	ICER £/ (QALY)		9,988
Cost per patient/10 years £	4,211	4,281	Cost per patient/19 years £	6,893	6,943

Incremental Cost £		70	Incremental Cost £		50
Effect per patient /10 years (QALYs)	5.82665	5.82990	Effect per patient /19 years (QALYs)	9.60334	9.60875
Incremental Effect (QALYs)		0.00326	Incremental Effect (QALYs)		0.00541
ICER £/ (QALY)		21,343	ICER £/ (QALY)		9,258
Cost per patient/11 years £	4,552	4,619	Cost per patient/20 years £	7,143	7,191
Incremental Cost £		67	Incremental Cost £		48
Effect per patient /11 years (QALYs)	6.30629	6.30982	Effect per patient /20 years (QALYs)	9.95527	9.96088
Incremental Effect (QALYs)		0.00353	Incremental Effect (QALYs)		0.00561
ICER £/ (QALY)		18,990	ICER £/ (QALY)		8,605

Supplementary Table8: Incremental cost-effectiveness ratios (ICERs) in different time horizon years for the UK population

ICER: incremental cost-effectiveness ratio, QALY: quality-adjusted life year.
