# Supplementary material for: Cost effectiveness of pharmacogenetic-guided clozapine administration based on risk of HLA variants in Japan and the UK
Source: Transl Psychiatry. 2021 Jul 7;11:362. doi: 10.1038/s41398-021-01487-4 (PMC8260588; doi:10.1038/s41398-021-01487-4)
Supplement: Supplementary file 2 — Supplementary Figures [file 41398_2021_1487_MOESM2_ESM.pdf]

(A)

Current estimated proportion of CIAG and HLA-B*59:01

	HLA-B*59:01 (+)	HLA-B*59:01 (-)	total
CIAG	1,280	4,160	5,440
CIAG (-)	2,681	91,879	94,560
Total	3,961	96,039	100,000

	HLA-B*59:01 (+)	HLA-B*59:01 (-)	Sub total
CIA	396 (~30%)	692	1,088
CIG	884 (~70%)	3,468	4,352
Sub total	1,280 (100%)	4,160	5,440

set "**CIAG prevention rate**" = 0.3

$1{,}280 \times 0.3 =$ **384**

if we prevent CIG in subjects with HLA-B*59:01,
a maximum ~70% CIAG prevention rate can be obtained.

Expected proportion by "HLA-guided treatment schedule"

	HLA-B*59:01 (+)	HLA-B*59:01 (-)	Sub total'
CIA	396	692	1088
CIG	**500**	3,468	**3,968**
Sub total	**896**	4,160	**5,056**

(~7.0% reduction)

(B)

Current estimated proportion of CIAG and HLA-B 158T/HLA-DQB1 126Q

	HLA-B 158T/HLA-DQB1 126Q(+)	HLA-B 158T/HLA-DQB1 126Q(-)	total
CIAG	1,235	2,195	3,430
CIAG (-)	10,623	85,947	96,570
Total	11,858	88,142	100,000

	HLA-B 158T/HLA-DQB1 126Q(+)	HLA-B 158T/HLA-DQB1 126Q(-)	Sub total
CIA	287 (~23%)	413	700
CIG	948 (~77%)	1,782	2,730
Sub total	1,235 (100%)	2,195	3,430

set "**CIAG prevention rate**" = 0.3

$1,235 \times 0.3 =$ **371**

if we prevent CIG in subjects with HLA-B 158T/HLA-DQB1 126Q(-)
, a maximum ~77% CIAG prevention rate can be obtained.

Expected proportion by "HLA-guided treatment schedule"

	HLA-B 158T/HLA-DQB1 126Q(+)	HLA-B 158T/HLA-DQB1 126Q(-)	Sub total'
CIA	287	413	700
CIG	**577**	1,782	**2,359**
Sub total	**658**	2,195	**3,059**

(~11% reduction)

Supplementary Fig. 1 CIAG prevention rate

(A) for Japanese population and (B) for the UK population.

CIAG: clozapine-induced agranulocytosis/granulocytopenia, CIA: clozapine-induced agranulocytosis, CIG: clozapine-induced granulocytopenia.

a maximum ~70% CIAG prevention rate can be obtained using Bayes' theorem:

e.g. Japanese population

$$\mathrm{Probablity(CIA|HLA-B*59:01)} = \frac{\mathrm{Probability(CIA)} \times \mathrm{Probability(HLA-B*59:01|CIA)}}{\mathrm{Probability(HLA-B*59:01)}}$$

$$= \frac{\text{Prevalence(CIA)} \times \text{"sensitivity for CIA"}}{\text{phenotype frequency of HLA-B*59:01(discussed below)}} = \frac{0.01088 \times 0.364}{0.0396} = 0.10$$

$$Probablity(CIG|HLA-B*59:01) = \frac{Probability(CIG) \times Probability(HLA-B*59:01|CIA)}{Probability(HLA-B*59:01)}$$

$$= \frac{Prevalence(CIG) \times \text{"}sensitivity\ for\ CIG\text{"}}{\text{phenotype frequency of HLA-B*59:01}(discussed\ below)} = \frac{0.04352 \times 0.293}{0.0396} = 0.223$$

thus,

$$Estimated\ proportion\ of\ CIG\ in\ CIAG\ for\ subjects\ with\ HLA-B*59:01$$

$$= \frac{0.223}{0.10 + 0.223} = 0.69$$

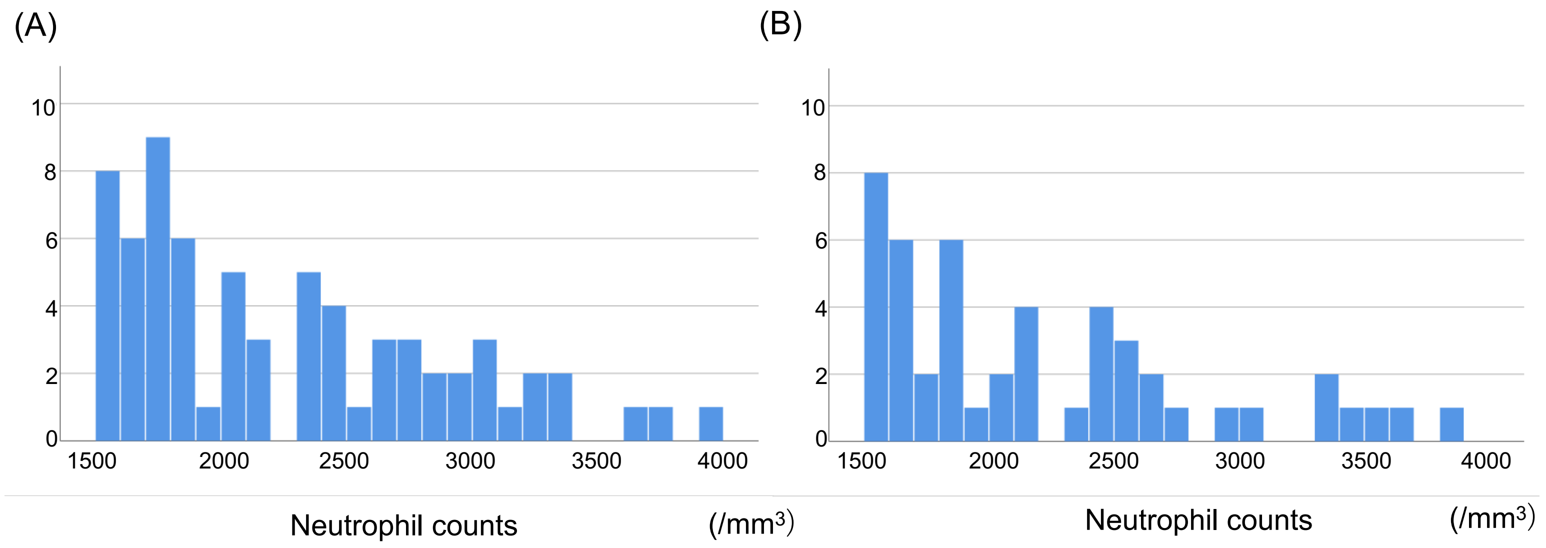

Supplementary Fig. 2 Absolute neutrophil counts (ANC) of CIAG subjects in Japan

(A) CIA, (B) CIG. X axis indicates bin for minimum neutrophil counts before onset of CIA or CIG (within six months after clozapine initiation) and Y axis indicates number of subjects.

CIAG: clozapine-induced agranulocytosis/granulocytopenia, CLZ: clozapine

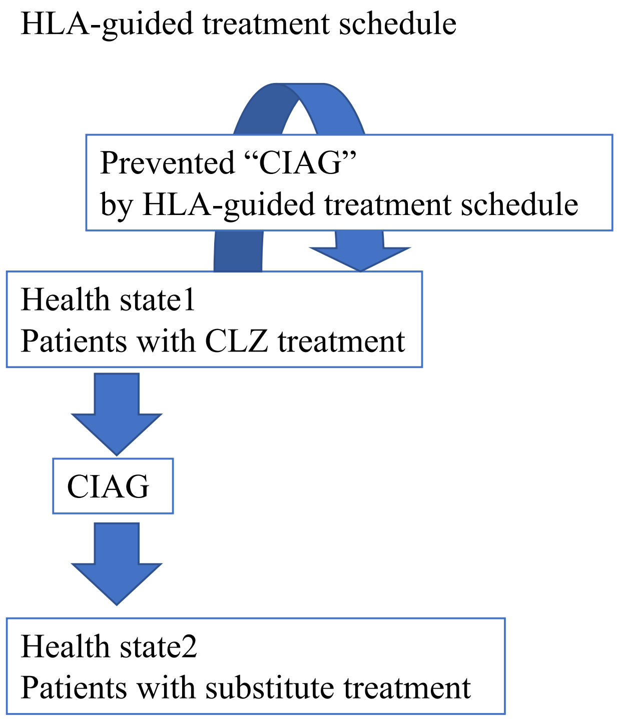

Supplementary Fig. 3 Markov model schematic

CIAG: clozapine-induced agranulocytosis/granulocytopenia, CLZ: clozapine

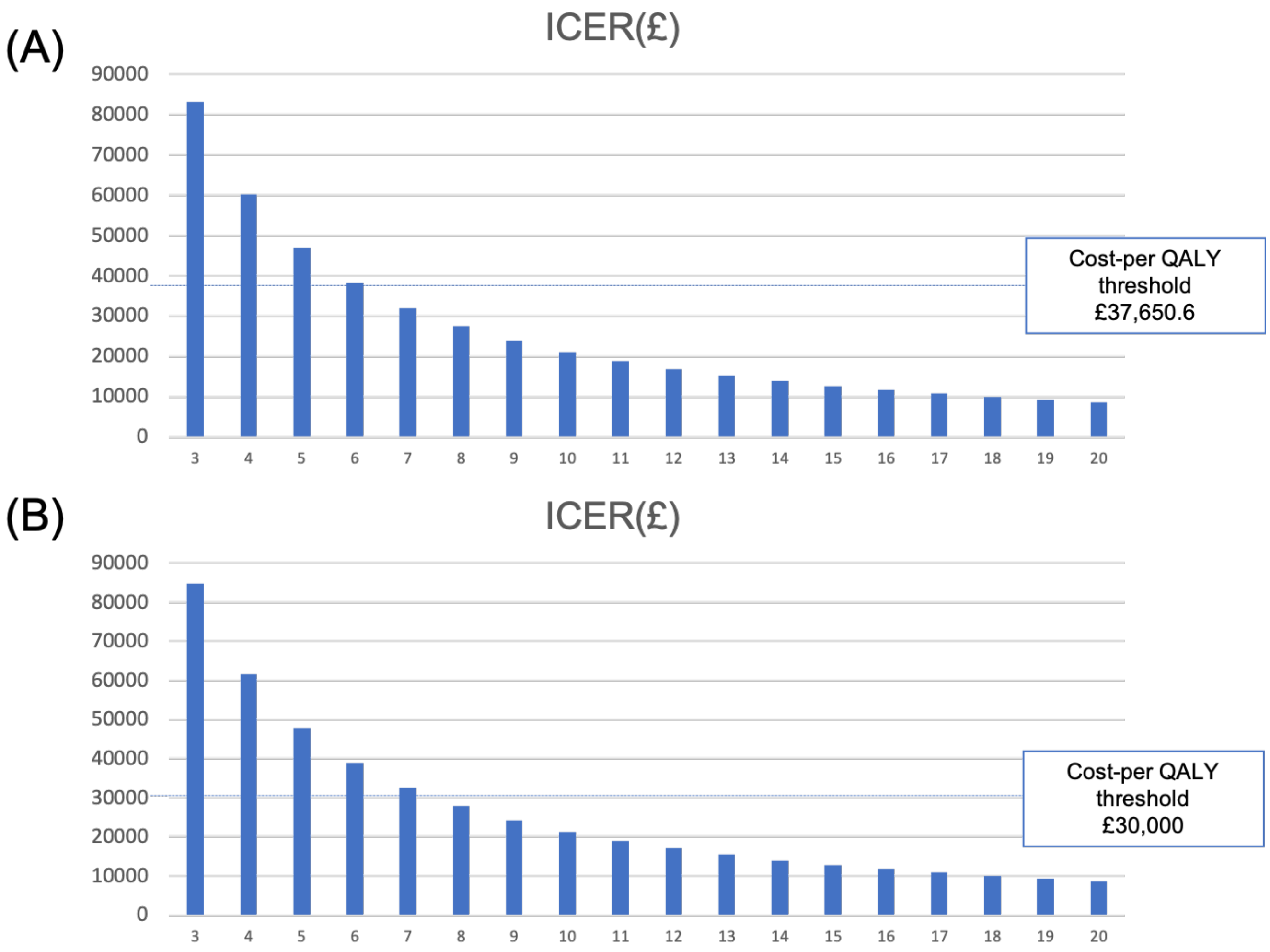

Supplementary Fig. 4 Relationship between the incremental cost-effectiveness ratios (ICERs) and time-horizons
